# Arthroscopic Bony Bankart Repair Using Double-Threaded Headless Screw: A Case Report

**DOI:** 10.1155/2012/789418

**Published:** 2012-01-11

**Authors:** Takeshi Kokubu, Issei Nagura, Yutaka Mifune, Masahiro Kurosaka

**Affiliations:** ^1^Department of Orthopaedic Surgery, Kobe University Graduate School of Medicine, Kobe 650-0017, Japan; ^2^Department of Orthopaedic Surgery, Kobe Rosai Hospital, Kobe 651–0053, Japan

## Abstract

We present a case of arthroscopic fixation for bony Bankart lesion using a double-threaded cannulated screw. A 39-year-old man sustained a left shoulder injury from a motorcycle accident. Radiographs showed bony Bankart lesion and CT revealed 40% defect of glenoid articular surface. Arthroscopic fixation was performed using double-threaded cannulated screw after the bony fragment was reduced by suturing the labrum at the edge with a suture anchor. Arthroscopic bony Bankart repair using double-threaded cannulated screw fixation is effective because compression force could be applied between bony fragments and the screw head is not exposed in the glenohumeral joint.

## 1. Introduction

Bankart lesions caused by traumatic glenohumeral dislocation are the avulsed capsulolabral structure to the glenoid rim [1]. Glenoid rim fractures which are often associated with Bankart lesions are called bony Bankart lesions and have the potential to result in persistent glenohumeral joint instability if the fragment is displaced or collapsed [2, 3]. Griffith et al. described that bony Bankart lesions were present in 16% of first shoulder dislocations and 23% of recurrent dislocations [4]. A bony defect with a width that is at least 21% of the glenoid length causes instability and limits the range of motion of the shoulder after Bankart repair of the capsulolabral structure [5].

Therefore, larger bony fragments should be treated by anatomical reduction and internal fixation. Several procedures have been reported to treat bony Bankart lesions by arthroscopic techniques [2, 6–11]. We present an arthroscopic bony Bankart repair using a double-threaded headless screw, which can achieve firm compression between the bony fragment and the glenoid.

## 2. Case Presentation

A 39-year-old man sustained a left shoulder injury from a motorcycle accident. He perceived a severe pain and deformity on his left shoulder immediately after the injury, however, this symptom decreased after a spontaneous reduction while lifting his left arm.

**Figure 1:**
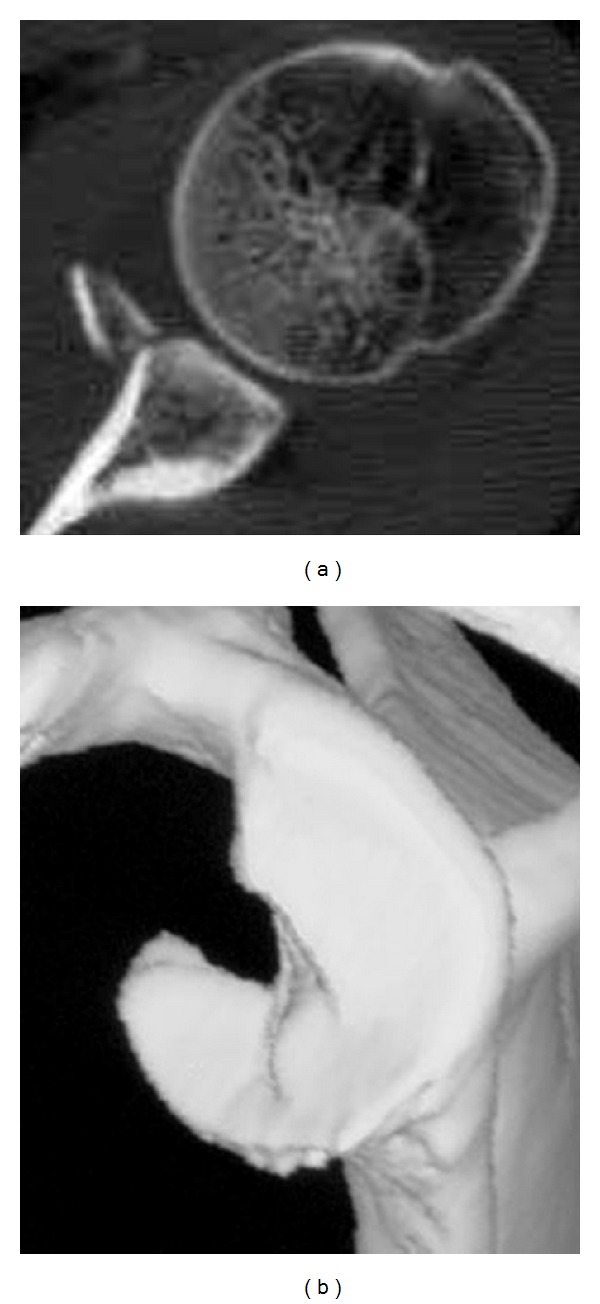
CT imagingat the time of injury. Axial image of CT shows a bony Bankart lesion at the anteroinferior part of glenoid articular surface (a). Size of the bony fragment was 17 mm × 27 mm and its width was 41% of the glenoid length on the 3-dimensional CT (b).

Plain radiographs in AP view showed a bony fragment at the inferior part of the glenoid. Computed tomography (CT) revealed a bony Bankart lesion at the anteroinferior part of glenoid articular surface (Figure 1(a)). Size of the bony fragment was 17 mm × 27 mm and its width was 41% of the glenoid length on the 3-dimensional CT (3D CT) evaluation (Figure 1(b)). We, therefore, decided to perform arthroscopic reduction and internal fixation of bony Bankart lesion using double-threaded headless screw.

**Figure 2:**
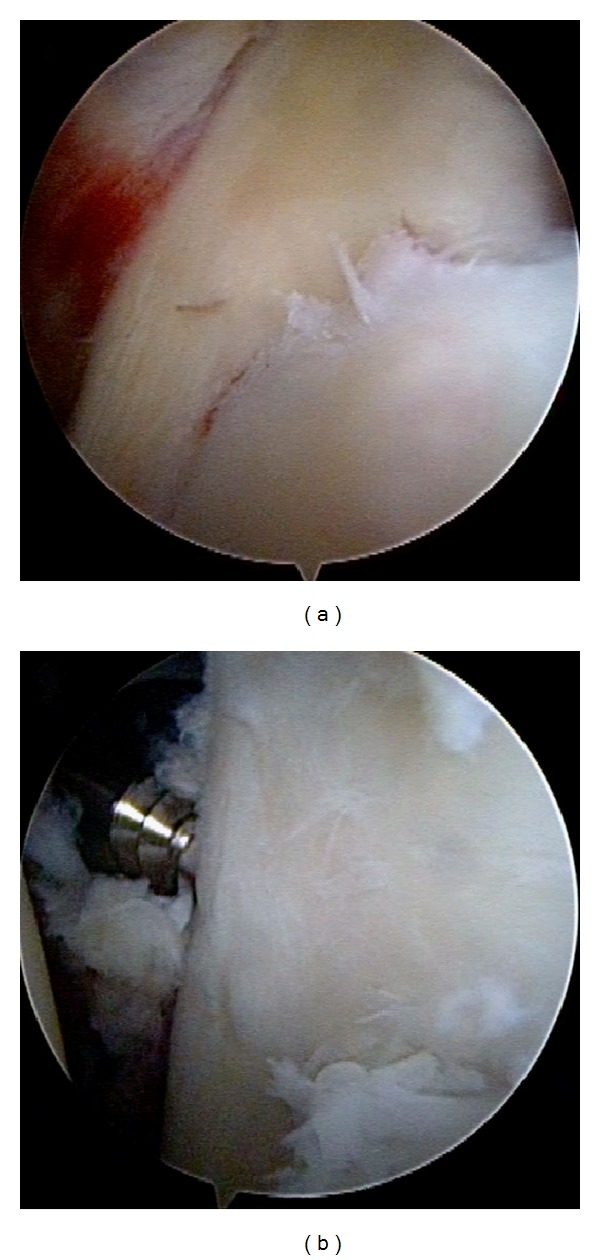
Arthroscopic view from the posterior portal. The bony fragment (F) could be easily reduced drawing up by grasper through the anterior portal (a). The bony Bankart lesion was fixed by DTJ screw after confirmation of flat reduction at the articular surface (b). G: glenoid.

The patient was placed in the beach-chair position under general anesthesia. Manual examination of his left shoulder confirmed full ROM and marked anteroinferior instability which caused a dislocation of the shoulder joint. Contralateral right shoulder showed no instability, which indicated the left shoulder instability caused by a traumatic injury. Arthroscope was introduced to the glenohumeral joint through the posterior portal. The displaced large bony fragment was found medially at the anteroinferior fracture rim of the glenoid. The capsulolabral structure was ruptured at the superior edge (9 o'clock) of the fragment. However, the labrum was attached to the fragment without avulsion and the cartilage surface at the inferior edge (6 o'clock) of the fragment continued to the glenoid rim. The bony fragment could be easily reduced drawing up by grasper through the anterior portal (Figure 2(a)) and the ruptured capsulolabrum at the superior part was repaired with conventional suture anchor technique. Thereafter, arthroscopic internal fixation using Double-threaded Japan (DTJ) screw (Meira, Japan) was performed. An anteroinferior portal was newly created at 7 o'clock through the subscapuralis muscle. A 1.2 mm guide wire was inserted to the bony fragment using a cannula and the bony Bankart lesion was fixed by 24 mm DTJ screw after confirmation of flat reduction at the articular surface (Figure 2(b)). The length of the screw was measured by CT imaging before the surgery.

**Figure 3:**
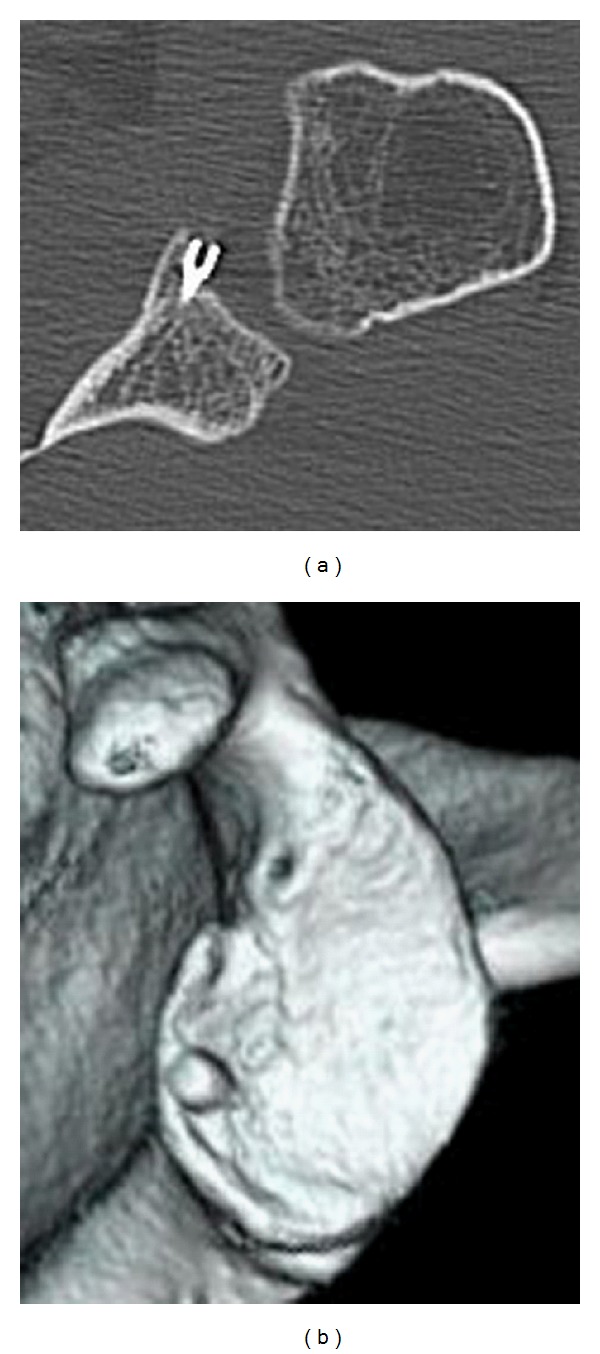
CT imaging at the time of revision. Axial image of CT showed union of the bony Bankart fragment, however, the screw end appeared to protrude intraarticular space (a). 3-dimensional CT (b).

**Figure 4:**
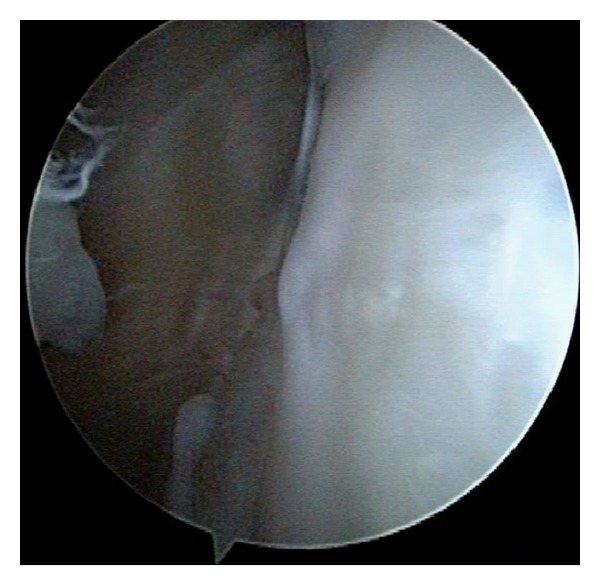
Second-look arthroscopy reveals the smooth articular surface at the bony Bankart lesion, and the screw end could not be found as existed under the articular cartilage.

 The patient's extremity was placed in a sling after surgery. Passive ROM exercise was allowed at postoperative 3 weeks, and active exercise was begun at 5 weeks after surgery. The patient had no apprehension and restriction of ROM at one year after surgery. CT showed union of the bony Bankart fragment, however, the screw end appeared to protrude intraarticular space (Figure 3). Since there was a possibility of screw impingement to the humeral head, a second-look arthroscopy was carried out. It revealed the smooth articular surface at the bony Bankart lesion, and the screw end could not be found as placed under the articular cartilage (Figure 4). However, there was a possibility that a prominent ridge of the screw head might be beneath the articular surface, therefore, the screw was removed.

## 3. Discussion

Bony Bankart lesions have been generally treated by open reduction and internal fixation [2, 12]. Recently, arthroscopic reduction and internal fixation of bony fragment using cancellous screws [6, 11] or suture anchors [7, 8, 10] has resulted in successful outcomes in terms of the recurrence rate and function in shoulders. The cancellous screw fixation provides a firm compression between the fragment and glenoid, however, the screw head is exposed intraarticular space which might impinge the humeral head in the [9, 10]. The conventional suture anchor repair does not provide compression of the fractured fragment, and the bony piece may tilt because of the single point fixation [8] To increase the contact area between the fragment and the glenoid, penetrating the fragment to pass a suture [10] or fixing the fragment with dual row technique has been introduced [7, 8], however, these methods are technically difficult. We choose DTJ screw, which is cannulated and headless, for arthroscopic reduction and internal fixation for the case in which fragment is larger than 10 mm in width, and this method enables to reduce the bony fragment easily and provides sufficient compression to the fracture site. Sano et al. [9] reported an arthroscopic treatment of an anterior glenoid fracture using the DTJ screw with suture anchors resulting in the good clinical result.

DTJ screw was modified from an original Herbert screw for scaphoid fractures, which is cannulated and can be used as thick as 1.2 mm guide wire [13]. This screw is headless and self-drilling which needs no removal of the screw if the screw is placed under the articular cartilaginous surface. A second-look arthroscopy of this case at postoperative 1 year revealed the healed smooth articular surface at the bony Bankart lesion although the screw end appeared to protrude over the articular cartilage on CT scan. The DTJ screw should be carefully placed deeply enough under the articular surface.

Arthroscopic bony Bankart repair using double-threaded cannulated screw fixation is effective because compression force could be applied between bony fragments and the screw head is not exposed in the glenohumeral joint. Reduction and internal fixation using DTJ screw in large bony Bankart lesion ensure an anatomical articular surface healing and an excellent functional outcome.

## 4. Consent

Consent was obtained from the patient for publication of this report and accompanying image.
